# No effect of Bt Cry1Ie toxin on bacterial diversity in the midgut of the Chinese honey bees, *Apis cerana cerana* (Hymenoptera, Apidae)

**DOI:** 10.1038/srep41688

**Published:** 2017-01-31

**Authors:** Hui-Ru Jia, Ping-Li Dai, Li-Li Geng, Cameron J. Jack, Yun-He Li, Yan-Yan Wu, Qing-Yun Diao, James D. Ellis

**Affiliations:** 1 Ministry Key Laboratory of Pollinating Insect Biology, Institute of Apicultural Research, Chinese Academy of Agricultural Sciences, Beijing 100093, China; 2 State Key Laboratory for Biology of Plant Diseases and Insect Pests, Institute of Plant Protection, Chinese Academy of Agricultural Sciences, Beijing 100193, China; 3 Honey Bee Research and Extension Laboratory, Department of Entomology and Nematology, University of Florida, Gainesville, Florida 32611, USA

## Abstract

Cry1Ie protein derived from *Bacillus thuringiensis* (Bt) has been proposed as a promising candidate for the development of a new Bt-maize variety to control maize pests in China. We studied the response of the midgut bacterial community of *Apis cerana cerana* to Cry1Ie toxin under laboratory conditions. Newly emerged bees were fed one of the following treatments for 15 and 30 days: three concentrations of Cry1Ie toxin (20 ng/mL, 200 ng/mL, and 20 μg/mL) in sugar syrup, pure sugar syrup as a negative control and 48 ng/mL imidacloprid as a positive control. The relative abundance of 16S rRNA genes was measured by Quantitative Polymerase Chain Reaction and no apparent differences were found among treatments for any of these counts at any time point. Furthermore, the midgut bacterial structure and compositions were determined using high-throughput sequencing targeting the V3-V4 regions of the 16S rDNA. All core honey bee intestinal bacterial genera such as *Lactobacillus, Bifidobacterium, Snodgrassella*, and *Gilliamella* were detected, and no significant changes were found in the species diversity and richness for any bacterial taxa among treatments at different time points. These results suggest that Cry1Ie toxin may not affect gut bacterial communities of Chinese honey bees.

The application of Bt plants create considerable environmental and economic benefits in terms of drastic decreases in conventional insecticide use, thus increasing farmer incomes^1^,^2^,^3^. However, Bt plants still give rise to a considerable amount of public debate despite that these toxins have been used for years and numerous studies have supported their safety for both animals and the environment^4^,^5^. One major concern is the risk of valued non-target organisms feeding on or visiting the Bt plants^6^.

As the most important worldwide pollinator, honey bees play a significant role in maintaining biodiversity and economic development^7^. Because they can feed on pollen from genetically modified (GM) plants expressing toxins, honey bees are exposed to the insecticidal protein^8^. Therefore, the honey bee has been recognized as a crucial non-target organism in the environmental risk assessment process for Bt plants^9^. Honey bee feeding tests with Cry toxins have been performed extensively in laboratory and field settings. The majority of studies focused on these parameters: bee longevity, consumption rates of treated food, development of hypopharyngeal glands, feeding and learning behavior, and superoxide dimutase activity. The evidences demonstrated no negative effect of Cry toxins on any of these parameters observed^10^,^11^,^12^,^13^,^14^,^15^,^16^,^17^,^18^,^19^.

Recently, accumulating evidence shows that intestinal microbiota plays a vital role in honey bee nutrient provisioning, pathogen defense and digestive efficiency, neutralization of dietary toxins and its balance is linked to health status of the host^20^,^21^,^22^. Thus, intestinal microbiota abundance and diversity have been used as parameters on which the impact of GM plants on many animals and honey bees have been tested^23^,^24^,^25^,^26^,^27^,^28^.

Cry1Ie is a novel toxin with a different insecticidal mechanism from that of other Bt toxins currently applied for pest control purposes. This may help to delay the evolution of insect resistance to Bt toxins^29^,^30^. Some investigators have confirmed that Cry1Ie may be a good candidate to be produced in new commercial Bt variety for pest control^31^,^32^,^33^. However, except for two recent studies that have assessed the effect of Cry1Ie toxin on Western honey bee (*Apis mellifera ligustica*) under laboratory conditions^18^,^28^, the impacts of Cry1Ie toxin on honey bees are poorly understood.

The Asian honey bee, *Apis cerana*, is a honey bee species indigenous to Asia and is the second most populous honey bee species in China^34^. Compared to western honey bees (*A. mellifera*) which are the most abundant bee species managed in China, *A. cerana* has many unique characteristics that make them useful pollinators; including better adaptability to local climate, disease resistance and the ability of utilizing scattered nectar^35^,^36^. Thus, proper risk assessment of any new Bt toxin on this species is indispensable, especially for China. However, studies evaluating the effects of Cry1Ie toxin on Chinese honey bees, *Apis cerana cerana*, are scarce. Here, we used midgut bacterial community as a parameter to identify the impacts of Cry1Ie toxin on Chinese honey bees, *Apis cerana cerana*, through next-generation sequencing technology on the MiSeq platform.

## Results

### Changes in bacterial 16S rRNA gene abundance

**Figure 1:**
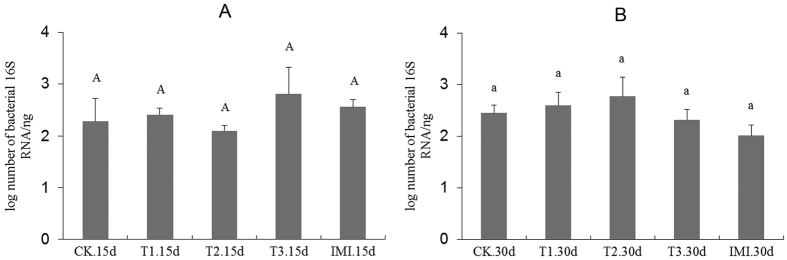
Log-transformed abundance of the 16S rRNA gene across different treatments at two sampling times. CK - Pure sugar syrup, T1–20 ng/mL Cry1Ie toxin syrups, T2–200 ng/mL Cry1Ie toxin syrups, T3–20 μg/mL Cry1Ie toxin syrups; IMI–48 ng/mL imidacloprid syrups. Bars with the same letter are not statistically different at α ≤ 0.05 as determined by Tukey’s HSD analysis.

The bacterial abundance was characterized by calculating 16S rRNA gene copy numbers using an absolute quantification qPCR method. For quantifying the copy numbers, a standard curve of y = −0.3628x + 10.3199 (y = the logarithm of plasmid copy number to base 2, x = Ct value, R^2^ = 0.995) was established as shown in Fig. S2. The copy number of 16S rRNA gene in each sample was calculated based on the standard curve (Table S1), then log-transformed prior to statistical analysis to satisfy the normality assumptions. The log10-transformed the copy numbers across different treatments are shown in Fig. 1, and these values were not significantly different among different treatments at any sample time (P > 0.05, Table S2). These results suggest that dietary Cry1Ie toxin has no significant impact on honey bee midgut bacterial abundance.

### Intestinal bacterial communities of adult Chinese honey bees

In the current study, we characterized the Chinese honey bee midgut bacterial community via 16S rRNA amplicons sequencing on Illumina MiSeq platform. Paired-end sequencing of 16S rRNA V3-V4 gene produced a total of 2,071,950 raw sequences from 30 samples. After trimming the barcodes, primers and filtering chimeras, short and low-quality reads, 53,372 effective sequences were obtained, ranging from 29,264 to 64,739 per sample, and the average length of effective sequences reads was 419 bp (Table S3). All valid sequences were normalized for further analysis. However, rarefaction analyses showed that the number of observed species did not approach saturation (Fig. S3), even after 30,000 sequences, which means that more sequencing efforts were needed to capture more species.

**Figure 2:**
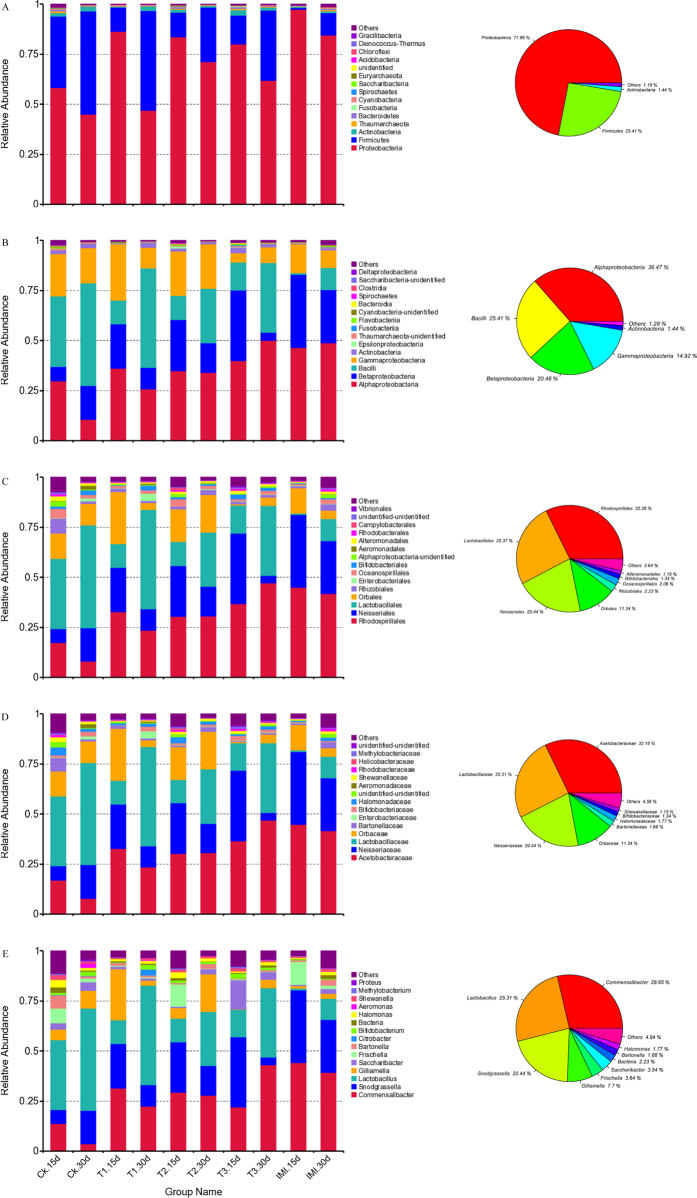
Relative abundance of the dominant midgut bacterial communities in Chinese honey bees (*Apis cerana cerana*) at phylum (**A**), class (**B**), order (**C**), family (**D**), and genera (**E**) levels. Each bar represents the average relative abundance of each bacterial taxon within a group. CK - Pure sugar syrup, T1–20 ng/mL Cry1Ie toxin syrups, T2–200 ng/mL Cry1Ie toxin syrups, T3–20 μg/mL Cry1Ie toxin syrups; IMI–48 ng/mL imidacloprid syrups.

To identify the phylogenetic diversity of midgut bacterial communities in Chinese honey bees, all effective reads were classified into different taxonomies (phylum, class, order, family, and genera levels) according to the QIIME using default settings, and the taxonomic distribution at different levels were summarized in Fig. 2. Overall, these bacterial taxa were dominant in all treatments (>1%): 3 phyla (Proteobacteria, Firmicutes and Actinobacteria), 5 classes (Alphaproteobacteria, Betaproteobacteria, Gammaproteobacteria, Bacilli, and Actinobacteria), 6 orders (Rhodospirillales, Lactobacillales, Neisseriales, Orbales, Rhizobiales, and Enterobacteriales), 7 families (Acetobacteraceae, Neisseriaceae, Lactobacillaceae, Orbaceae, Bartonellaceae, Enterobacteriaceae, and Bifidobacteriaceae), and 8 genera (*Commensalibacter, Lactobacillus, Snodgrassella, Gilliamella, Frischella, Saccharibacter, Bifidobacterium* and *Bartonella*).

Based on the average relative abundance, Proteobacteria (70%) was the most abundant phyla among the classified bacterial phyla. The classes with the highest abundance of bacteria were α-Proteobacteria (36.47%), Bacilli (25.41%), β-Proteobacteria (20.48%), and γ-Proteobacteria (14.92%). *Commensalibacter* of the family of Acetobacteraceae (28.65%), followed by *Lactobacillus* (25.31%), *Snodgrassella* (20.44%), were the 3 most abundant genera found.

### Effects of the Bt Cry1Ie toxin on the midgut bacterial composition of Chinese honey bees

**Figure 3:**
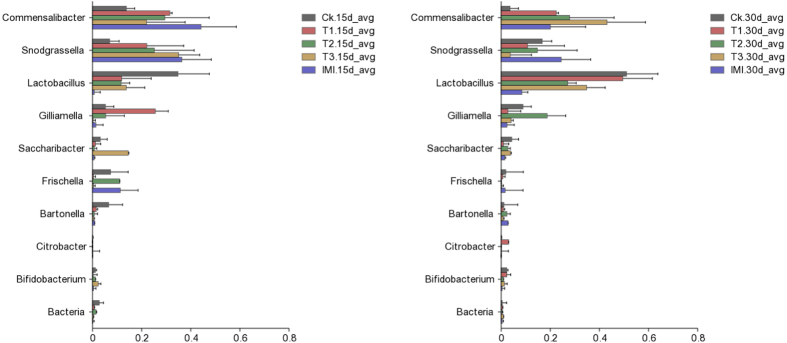
The relative abundance of the 10 most abundant bacteria genera in the midgut of Chinese honey bee (*Apis cerana cerana*) adults across the five treatments at two sampling times. Horizontal bars represent the standard error of the means composed of three replicates.

To determine the changes in the midgut bacterial communities of Chinese honey bees fed Cry1Ie toxin, a statistical analysis for the relative abundances of the 10 most abundant genera were conducted using one-way ANOVA (SPSS. 16.0). Among the 10 most abundant bacteria genera were *Commensalibacter, Lactobacillus, Snodgrassella, Gilliamella, Frischella, Saccharibacter, Bartonella, Bifidobacterium, Citrobacter*, and *Bacteria*. All were present in all treatment bee groups as major genera (>1%) (Table S4), and the histograms of these bacterial taxa are shown in Fig. 3. An analysis on the 10 most abundant genera revealed no significant differences in the relative abundances of any bacterial taxon with respect to the different treatments (Table S5, P > 0.05).

**Figure 4:**
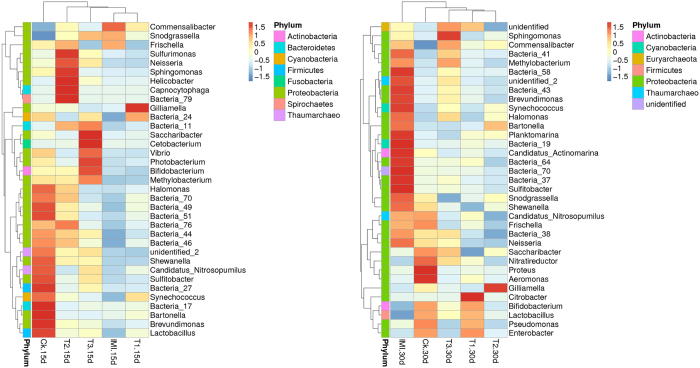
Hierarchically clustered heat map analysis of the highly represented bacterial taxa (at the genus level) found in the midgut of Chinese honey bee (*Apis cerana cerana*) workers (relative abundance > 1%) across the five treatments at two sampling times. The relative percentages (%) of the bacterial families are indicated by varying color intensities according to the legend at the top of the figure. The color key for the Z score indicates correspondence between blue-red coloring and standard deviations from the mean abundance of each bacteria. For those bacterial taxa unable to be assigned into specific known bacterial genera, the higher known taxonomic unit was added: Bacteria_11 (c_Actinobacteria); Bacteria_17 (f_Flammeovirgaceae); Bacteria_19 (f_Flavobacteriaceae); Bacteria_24 (o_Ignavibacteriales); Bacteria_27 (p_Cyanobacteria); Bacteria_37 (f_Gemmatimonadaceae); Bacteria_38 (c_Gemmatimonadetes); Bacteria_41 (c_Oligosphaeria); Bacteria_43 (c_Phycisphaerae); Bacteria_44 (Planctomycetaceae); Bacteria_46 (f_Hyphomonadaceae); Bacteria_49 (f_Hyphomonadaceae); Bacteria_51 (f_Acetobacteraceae); Bacteria_58 (f_Alcaligenaceae); Bacteria_64 (f_Rhodocyclaceae); Bacteria_70 (f_Sandaracinaceae); Bacteria_76 (f_Chromatiaceae); Bacteria_79 (f_Coxiellaceae).

Furthermore, the difference of the midgut microbial community composition in different treatments was evaluated further using Hierarchical cluster analysis. Hierarchically clustered heat maps of all the abundant genera (relative abundance >1%) are shown in Fig. 4. This figure shows that mostly all of the presented bacterial taxa here were clustered together corresponding to different treatments. These results demonstrate that the midgut bacterial composition across the different treatment groups was not significantly different.

### Effects of the Bt Cry1Ie toxin on the midgut bacterial structure of Chinese honey bees

**Figure 5:**
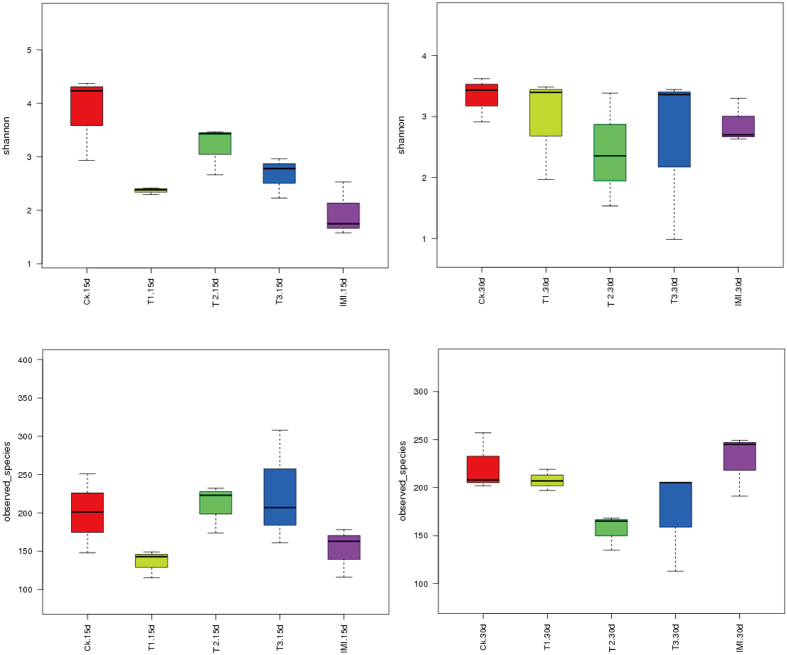
Box plot of mean alpha diversity for the five treatments at two sampling times. Error bars indicate standard errors.

To determine if Chinese honey bee midgut microbiota community structures were altered by the Cry1Ie toxin, the bacterial diversity was analyzed across different treatments. The bacterial diversity was characterized by calculating the alpha diversity parameters (Table S6). Two representatively alpha diversity parameters including observed species (sequencing depth) and the Shannon index (diversity indices), were selected for community richness comparison. Box plots of these richness estimators for individual sample groups are shown in Fig. 5. Comparison diversity indexes among groups were compared using a one-way ANOVA (SPSS. 16.0) with no significant differences in richness noticed across the five treatment groups (P > 0.05, Table S7).

**Figure 6:**
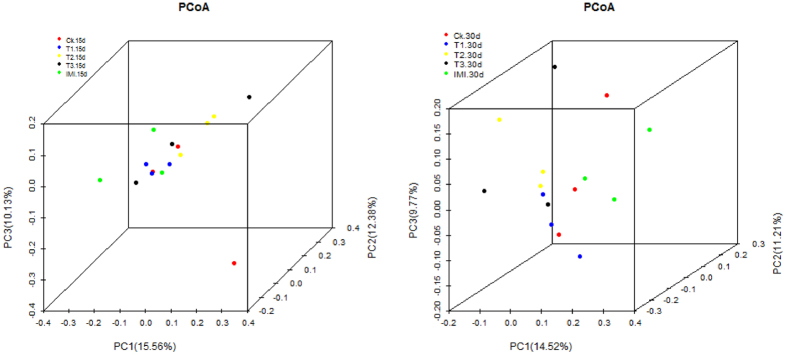
3-D score plot for the three principal components of the midgut microbiota among five treatments at two sampling times. Each symbol represents each sample gut microbiota.

For a better analysis of the relationships between gut microbiota community structures of the honey bees across the five treatments, the Principal Coordinate Analysis (PCoA) was performed based on the Unifrac metric. A 3-D plot of first principal component (PC1), second principal component (PC2), and third principal component (PC3) obtained by PCoA is shown in Fig. 6. The plot elucidated the characteristics of four botanical origins of the bacterial communities. The percentages are the percentage of total community variations explained by the components. On the PCoA plot, each symbol represents the gut microbiota of a sample. Sample points that are close together are more similar in community composition than those that are far apart. No significant differences in community structure were observed. The pattern revealed by PCoA were tested further using ANOSIM. Consistent with the PCoA plot, no significant impacts were noticed (Table S8). These data suggest that the midgut bacterial community structures in honey bees are not be influenced by the Bt Cry1Ie toxin.

## Discussion

The potential risk of Bt toxin on the intestinal microbiota on non-target organisms is a major safety concern and requires careful consideration. Several studies have addressed the impact of Bt Cry toxin on honey bee intestinal microbiota; however, investigators in these studies mainly adopted conventional molecular methods, such as terminal restriction fragment length polymorphism (T-RFLP)^23^,^27^ and polymerase chain reaction-denaturing gradient gel electrophoresis (PCR-DGGE)^25^,^26^ to investigate the impacts. Many studies claimed that these conventional molecular methods were considered specific, but low-throughput and low sensitivity in characterizing microbial ecology merely provides preliminary information. Thus, the use of these methods to investigate the changes in microbial community structure and diversity cannot provide comprehensive insights to their differences and may result in some bacteria remaining undetected. For instance, previous studies have reported that *Lactobacillus* plays a significant role in honey bee health and that this genus is one of the core microflora within honey bees^37^,^38^. However, when PCR-DGGE was used, *Lactobacillus* was not detected during a study on the influence of transgenic *cry1Ah* maize pollen on the midgut bacteria community of the Chinese honey bee^25^.

As molecular biological technology continues to develop, some burgeoning technologies such as high-throughput sequencing is being applied frequently to characterize microbial ecology, including in the honey bee intestinal microbiota^20^,^28^,^39^,^40^,^41^,^42^. These technologies have many benefits including high sensitivity, high accuracy and short processing times^43^,^44^. Moreover, these approaches have enough sequencing depth to cover complex bacterial communities, and the diversity in microbial populations is significantly higher than previously estimated by earlier conventional molecular methods^45^,^46^. In previous work, this method has been first applied into the Cry1Ie toxin risk assessment of another bee species *A.mellifera*^28^. In the current study, a 16S rRNA amplicons sequencing method was also used to examine the potential effects of Cry1Ie toxin on the midgut bacterial communities of Chinese honey bees. Our findings correspond with our previous reports that neither the midgut bacterial diversity nor their compositions were affected when the bees were exposed to Cry1Ie toxin^28^. These results suggest that Cry1Ie does not impact the gut bacterial communities of honey bees.

Honey bee intestinal microbiota has been extensively investigated using both culture-dependent and culture-independent methods. In this study, we also characterized the dominant midgut bacteria of Chinese honey bees through Illumina MiSeq paired-end sequencing of 16S rRNA gene, which were Commensalibacter, Lactobacillus, Snodgrassella, Gilliamella, Frischella, Saccharibacter, Bartonella, Bifidobacterium and Citrobacter from α-, β-, γ-Proteobacteria, Firmicutes and Actinobacteria. In agreement with previously published results^20^,^42^, all these conserved bacterial groups have been found in Chinese honey bees in our study. Reports indicate that adult honey bee worker guts are dominated by a few specific gut bacterial groups; merely the proportions of these phylotypes differ depending on diet and species^47^,^48^. For instance, a previous study investigated the intestinal microbiota of another bee species (*A.mellifera*), γ-Proteobacteria was the most common group of bacteria^28^; while, in this study, the most abundant group of bacteria was α-Proteobacteria.

Interestingly, the *Commensalibacter* was the most abundant bacterial genus in this study and its abundance was much higher than previously described. This genus belongs to the family Acetobacteraceae which is commonly found as commensal bacteria in many insects, including the honey bee^49^. Recent studies suggest this genus is a major intestinal symbiont involved in many key functions of the host, such as immunity, metabolism, and growth^49^,^50^,^51^,^52^. Earlier researchers have suggested that populations of this bacterial taxon rely on sugar-rich diets^49^, so the high levels of *Commensalibacter* in our study is probably related to the sugar diets fed to honey bees in the laboratory and may not accurately reflect the *Commensalibacter* levels found naturally in *A. cerana*.

In the current study, we used qPCR and high-throughput sequencing to assess the effects of Cry1Ie toxin on the midgut bacterial community of Chinese honey bees. No significant differences in the midgut bacterial abundance and composition were observed among the treatments and thus, we conclude that Cry1Ie does not affect bacterial diversity in the midguts of Chinese honey bees. Our work establishes a foundation leading to increased understanding of the effects of this toxin on honey bees and provides laboratory-based evidence for its future use in transgenic plants to control insect pests. However, laboratory research is merely the first step of the biosafety assessment of a transgenic crop^53^,^54^. In order to provide a comprehensive risk assessment, future work under more realistic conditions (i.e., bees fed on GM pollen or field trials) is needed.

## Methods

### Honey bees and Cry1Ie toxin

Honey bee queens were caged on a brood comb to achieve a uniform age. The combs containing nine-day old capped Chinese honey bee pupae, were brought from apparently healthy colonies located at the Department of Bee Protection and Biological Safety, Institute of Apicultural Research, Chinese Academy of Agricultural Sciences (CAAS), Beijing, China (40°00′28″N, 116°12′18″E). The brood combs were placed in the incubator at 34 ± 1 °C. All experimental work was conducted in June of 2015.

The Cry1Ie toxin used in the experiments was kindly provided by Prof. Jie Zhang from the State Key Laboratory for Biology of Plant Diseases and Insect Pests, the Institute of Plant Protection, CAAS, Beijing, China. The toxin was stored at −20 °C and mixed thoroughly with sugar syrup (60% w/v sucrose solution) to obtain the desired concentrations in our study (20 ng/mL, 200 ng/mL, and 20 μg/mL).

### Experimental design and laboratory feeding of honey bees

Newly emerged adult bees (<6 h old) were collected from these frames and randomly placed into wooden, mesh-sided cages (dimensions of 10 cm × 7 cm × 8 cm) (Fig. S1). Each cage contained 30 bees and all cages were randomly assigned to the different treatment groups and maintained in a dark incubator (30 ± 1 °C, 60 ± 10% relative humidity) until the end of the experiment. In the study, honey bees were exposed to Cry1Ie toxin via an artificial sucrose diet. Altogether, there were five defined diets which included pure sugar syrup (60% w/v sucrose solution, the negative control), three concentrations of Cry1Ie toxin (20 ng/mL, 200 ng/mL, and 20 μg/mL)^18^ and 48 ng/mL imidacloprid syrup, the positive control^55^. There were three replicates for each treatment. A surplus of pollen and treatment syrup were fed *ad libitum* and were replaced daily until further honey bee dissection and DNA extraction.

### Honey bee dissection and DNA extraction

After 15 and 30 d of exposure to treatments, 5 bees were randomly removed from each cage. These bees were placed in the freezer at −20 °C for 10 s to render them inactive, and their midguts were isolated on ice using sterile forceps. Dissected midguts from the same treatment group were pooled in a 2 mL eppendorf tube and immediately frozen in liquid nitrogen for subsequent DNA extraction.

The total midgut bacterial DNA from each sample was extracted with a QIAamp DNA Stool Mini Kit (Qiagen, catalogue number 51504, Hilden, Germany) according to the manufacturer’s protocol. The concentration and purity of the obtained DNA were evaluated using the Nanodrop ND 2000 (Nanodrop Technologies, Wilmington, DE, USA).

### Quantitative polymerase chain reaction (qPCR)

The bacterial 16S rRNA gene was quantified by qPCR on an ABI7500 PCR System (Applied Biosystems, Carlsbad, CA, USA) applying the universal bacterial primer pairs BAC27F/ BAC355R^56^. The qPCR reactions were performed in a 20 μL volume containing 10 μL 2x Es Taq MasterMix, 0.5 μL of each primer (10 μM), 1 μL of five-fold diluted template DNA template, and 8 μL of sterilized H_2_O. Amplification conditions were as follows: an initial denaturation of 3 min at 95 °C; five touch-down cycles of 20 s at 95 °C, 10 s from 65 °C to 60 °C, 20 s at 68 °C, followed by 38 cycles of 15 s at 95 °C, 15 s at 58 °C, and 20 s at 68 °C. In order to avert technical error, each qPCR reaction for all test samples was performed in triplicate. Standard curves were constructed from 10-fold serial dilutions of plasmid containing the cloned target genes. 16S rRNA gene copy numbers in test samples were calculated by linear regression of the normalized sample Ct values to the standard curve.

### MiSeq sequencing of 16S rRNA gene amplicons

The V3-V4 region of the bacterial 16S rRNA gene was targeted with the barcoded primer pair 341 f/806r (341 F: CCTAYGGGRBGCASCAG, 806 R: GGACTACNNGGGTATCTAAT) for the microbial community diversity analysis^57^,^58^. All PCR reactions in a 30 μL mixture contained 15 μL 2× Phusion Master Mix (New England Biolabs, USA), 1 μL of forward and reverse primers, and about 10 ng DNA template. Amplification conditions were as follows: initial denaturation at 98 °C for 1 min, followed by 35 cycles of denaturation at 98 °C for 10 s, annealing at 50 °C for 30 s, extension at 72 °C for 30 s, and a final extension step at 72 °C for 5 min. The PCR products were checked by electrophoresis on 2% agarose gels and purified with GeneJET Gel Extraction Kit (Thermo Scientific). Finally, according to protocols described by Caporaso^58^, the purified products were sequenced on the Illumina MiSeq 250 platform (Illumina, San Diego, CA, USA) at Novogene Bioinformatics Technology Co., Ltd, Beijing, China.

### Data analysis

Paired-end reads were assigned to samples according to the unique barcode of each sample. After cutting off the barcode and primer sequence, the reads that were shorter than twice the length of reads were merged into single, longer sequences using FLASH v1.2.7 (http://ccb.jhu.edu/software/FLASH/)^59^. The splicing sequences were called “raw reads”. The low-quality sequences (shorter than 200 bp, average quality value of < 25) and the chimera sequences then were filtered out from downstream analysis using QIIME V1.7.0 software package (http://qiime.org/index.html)^60^ and UCHIME algorithm (http://www.drive5.com/usearch/manual/uchime_algo.html)^61^ with the default parameters. The obtained sequences were called “clean reads” and normalized to make the samples compared at the same sequencing depth for the following analysis.

These normalized sequences were classified into operational taxonomic units (OTUs) at 97% similarity using UPARSE pipeline v7.0.1001 (http://drive5.com/uparse/)^62^. The taxonomy of the OTUs was assigned by blasting against SILVA SSU database 119^63^,^64^ with default parameters. Alpha diversity included Shannon index and observed species of each sample and beta diversity included both unweighted and weighted Unifrac distances between samples, were performed with QIIME (Version 1.7.0) and displayed with R software (Version 2.15.3).

### Statistical analysis

To determine whether the composition and structure of midgut bacterial communities differed significantly among treatments, statistical comparisons were made across different treatments on the 16S rRNA gene copy numbers, the dominant midgut bacterial genera composition and the richness estimators. The bacterial counts and relative abundance values were normalized with log-transformations to the base 10 prior to statistical analysis. All statistical analyses were conducted between each treatment with one-way analysis of variance (ANOVA) followed by Tukey’s HSD using the software package SPSS 16.0 (IBM Co., Armonk, NY, USA).

## Additional Information

**Accession codes**: Raw sequencing data retrieved in this study have been submitted to the National Center for Biotechnology Information (NCBI) Sequence Reads Archive (SRA) under accession number SRP091743.

**How to cite this article**: Jia, H.-R. *et al*. No effect of Bt Cry1Ie toxin on bacterial diversity in the midgut of the Chinese honey bees, *Apis cerana cerana* (Hymenoptera, Apidae). *Sci. Rep.* **7**, 41688; doi: 10.1038/srep41688 (2017).

**Publisher's note:** Springer Nature remains neutral with regard to jurisdictional claims in published maps and institutional affiliations.

## Supplementary Material

Supplementary Information
