# Supplementary material for: No effect of Bt Cry1Ie toxin on bacterial diversity in the midgut of the Chinese honey bees, Apis cerana cerana (Hymenoptera, Apidae)
Source: Sci Rep. 2017 Jan 31;7:41688. doi: 10.1038/srep41688 (PMC5282592; doi:10.1038/srep41688)

No effect of Bt Cry1Ie toxin on bacterial diversity in the midgut of the Chinese honey bees, *Apis cerana cerana* (Hymenoptera, Apidae)

Hui-Ru Jia[1, 2], Ping-Li Dai[1]*, Li-Li Geng[2], Cameron J. Jack[3], Yun-He Li[2], Yan-Yan Wu[1], Qing-Yun Diao[1], James D. Ellis[3]

1. Ministry Key Laboratory of Pollinating Insect Biology, Institute of Apicultural Research, Chinese Academy of Agricultural Sciences, Beijing 100093, China

2. State Key Laboratory for Biology of Plant Diseases and Insect Pests, Institute of Plant Protection, Chinese Academy of Agricultural Sciences, Beijing 100193, China

3. Honey Bee Research and Extension Laboratory, Department of Entomology and Nematology, University of Florida, Gainesville, Florida 32611, USA

*Corresponding author. Tel. (fax): +86 10 62597285. E-mail address: daipingli@caas.cn.

Additional information

Table S1 16S rRNA gene copy numbers per sample.

Table S2 Statistical analysis of the copy number of 16S rRNA gene of each treatment by one-way ANOVA (SPSS. 16.0).

Table S3 OUT's data. Data were obtained using the Illumina Miseq2500-pyrosequencing.

Table S4 The 10 most abundant bacterial genera within each sample.

Table S5 Statistical analysis of the composition of the 10 most abundant midgut bacterial genera among different groups by one-way ANOVA (SPSS. 16.0).

Table S6 Richness estimator of 30 samples.

Table S7 Statistical analysis of richness estimator among different groups by one-way ANOVA (SPSS. 16.0).

Table S8 ANOSIM statistical analysis among different groups at 2 sampling time, respectively.

Fig. S1 Melting curve, amplification curve, and standard curve of qPCR.

Fig. S2 Rarefaction on species-abundance data. Average value of 3 replicates is shown.

Fig. S3 Wooden cage and feeder.

Table S1 16S rRNA gene copy numbers per sample.

Sample name	copy number (copies/ng)
CK.15d.1	1.46E+03
CK.15d.2	6.07E+01
CK.15d.3	8.04E+01
T1.15d.1	4.32E+02
T1.15d.2	1.56E+02
T1.15d.3	2.60E+02
T2.15d.1	9.73E+01
T2.15d.2	2.05E+02
T2.15d.3	1.04E+02
T3.15d.1	3.13E+02
T3.15d.2	1.38E+02
T3.15d.3	6.55E+03
IMI.15d.1	5.55E+02
IMI.15d.2	4.75E+02
IMI.15d.3	1.90E+02
CK.30d.1	2.83E+02
CK.30d.2	5.09E+02
CK.30d.3	1.52E+02
T1.30d.1	2.39E+02
T1.30d.2	1.30E+03
T1.30d.3	1.94E+02
T2.30d.1	1.58E+02
T2.30d.2	2.96E+03
T2.30d.3	4.34E+02
T3.30d.1	1.05E+02
T3.30d.2	5.09E+02
T3.30d.3	1.66E+02
IMI.30d.1	1.69E+02
IMI.30d.2	1.61E+02
IMI.15d.3	3.92E+01

Table S2 Statistical analysis of the copy number of 16S rRNA gene of each treatment by one-way ANOVA (SPSS. 16.0).

Group	df	F	P
CK.15d, T1.15d, T2.15d, T3.15d, IMI.15d	14	0.671	0.589
CK.30d, T1.30d, T2.30d, T3.30d, IMI.30d	14	1.05	0.413

Table S3 OUT's data. Data was obtained using the Illumina Miseq2500-pyrosequencing.

Sample Name	Raw PE (#)	Raw Tags (#)	Clean Tags (#)	Effective Tags	Base (nt)	Avglen (nt)
CK.15d.1	62,844	57,075	48,326	47,538	19,947,813	420
CK.15d.2	65,120	58,476	50,016	49,339	20,688,511	419
CK.15d.3	69,832	62,724	53,552	52,604	22,156,347	421
CK.30d.1	63,128	57,663	49,063	48,250	20,555,948	426
CK.30d.2	75,297	68,367	57,839	56,839	24,109,631	424
CK.30d.3	65,898	59,990	50,744	49,972	21,286,100	426
IMI.15d1	60,735	55,202	48,355	47,744	19,728,868	413
IMI.15d2	65,040	59,329	51,532	50,958	21,254,097	417
IMI.15d3	73,359	66,630	57,614	56,859	23,875,060	420
IMI.30d1	68,936	61,943	52,089	51,457	21,870,758	425
IMI.30d2	74,313	67,259	59,212	58,337	24,047,651	412
IMI.30d3	73,263	66,364	58,514	57,685	23,644,550	410
T1.15d.1	76,006	69,029	59,255	58,903	24,780,065	421
T1.15d.2	76,141	69,056	61,237	60,516	24,826,761	410
T1.15d.3	77,927	70,549	59,394	58,789	25,120,498	427
T1.30d.1	65,490	59,262	49,782	48,944	20,759,597	424
T1.30d.2	78,955	71,996	62,608	61,616	25,542,423	415
T1.30d.3	68,173	62,341	53,108	51,031	21,744,260	426
T2.15d.1	65,405	58,970	49,504	49,046	20,878,429	426
T2.15d.2	37,452	34,004	29,620	29,264	12,200,461	417
T2.15d.3	69,045	62,735	55,209	54,502	22,600,917	415
T2.30d.1	69,508	63,491	54,120	53,600	22,733,399	424
T2.30d.2	67,758	62,736	56,707	55,767	22,730,497	408
T2.30d.3	68,566	62,208	52,216	51,540	22,060,790	428
T3.15d.1	61,137	55,607	48,791	48,176	19,952,040	414
T3.15d.2	76,141	69,236	59,054	58,215	24,692,787	424
T3.15d.3	69,199	63,556	55,404	54,670	22,639,088	414
T3.30d.1	78,349	71,238	61,355	60,623	25,275,028	417
T3.30d.2	78,577	71,878	64,907	64,739	26,274,864	406
T3.30d.3	70,368	64,042	54,255	53,650	22,729,933	424

Table S4 The abundances of top10 classified bacterial genera within each sample.

	Commensalibacter	*Snodgrassella*	*Lactobacillus*	*Gilliamella*	*Saccharibacter*	*Frischella*	*Bartonella*	*Citrobacter*	*Bifidobacterium*	**other**
CK.15d.1	0.070466	0.024396	0.588643	0.005031	0.087454	0.001792	0.178939	0	0.021260	0.022019
CK.15d.2	0.165363	0.037421	0.301092	0.118879	0.002447	0.002653	0.005789	0	0.005651	0.360704
CK.15d.3	0.178147	0.147066	0.154268	0.034458	0.005100	0.216085	0.013094	0.004032	0.007753	0.239999
CK.30d.1	0.020778	0.467179	0.272458	0.068985	0.033424	0.030599	0.022398	0.000793	0.004686	0.078702
CK.30d.2	0.034837	0.019434	0.661555	0.008546	0.084077	0.00162	0.005927	0.00124	0.055064	0.127701
CK.30d.3	0.052858	0.013576	0.596327	0.187967	0.011130	0.024603	0.004273	0.001413	0.008477	0.099376
T1.15d.1	0.30757	0.086558	0.169567	0.399917	0.003273	0.003549	0.001999	0	0.005513	0.022053
T1.15d.2	0.631784	0.033114	0.051135	0.143241	0.032700	0.003790	0.042693	0	0.000965	0.060577
T1.15d.3	0.005238	0.543503	0.134971	0.224148	0.000413	0.003790	0.001103	6.89E-05	0.001826	0.084938
T1.30d.1	0.110368	0.279522	0.471486	0.022570	0.012646	0.000517	0.015368	0.001516	0.027670	0.058337
T1.30d.2	0.533648	0.021329	0.377795	0.013714	0.01206	0.000551	0.003653	0.001172	0.001551	0.034527
T1.30d.3	0.028841	0.021123	0.636436	0.044795	0.002653	0.020571	0.007167	0.087557	0.038799	0.112057
T2.15d.1	0.020399	0.435788	0.159746	0.111574	0.001309	0.079356	0.007443	0.000207	0.031357	0.152820
T2.15d.2	0.355777	0.292685	0.114193	0.017815	0.006099	0.004238	0.006202	0	0.001034	0.201957
T2.15d.3	0.505944	0.026222	0.075532	0.030667	0.013990	0.249750	0.006926	0	0.002550	0.088419
T2.30d.1	0.053306	0.370766	0.339306	0.02033	0.075600	0.001137	0.004376	0.000965	0.021260	0.112953
T2.30d.2	0.772475	0.005754	0.121498	0.014231	0.003928	0.000551	0.063954	0.000551	0.001447	0.015609
T2.30d.3	0.010544	0.06516	0.351194	0.52717	0.001413	0.001378	0.001964	0.000896	0.003963	0.036319
T3.15d.1	0.513283	0.34096	0.035939	0.00348	0.008373	0.008821	0.006685	0	0.001895	0.080562
T3.15d.2	0.031736	0.692154	0.017367	0.002447	0.029255	0.001826	0.000758	0	0.002826	0.221633
T3.15d.3	0.115606	0.018538	0.35812	0.001999	0.398125	0.001964	0.006547	0	0.065091	0.034010
T3.30d.1	0.389683	0.102753	0.326281	0.036319	0.003239	0.003411	0.006650	0.001413	0.025016	0.105234
T3.30d.2	0.883188	0.002067	0.065401	0.000965	0.007684	6.89E-05	0.000861	0.000103	0.000276	0.039385
T3.30d.3	0.019951	0.006581	0.65239	0.083629	0.102925	0.004721	0.017436	0.000551	0.014782	0.097033
IMI.15d.1	0.593846	0.353261	0.008546	0.000930	0.007340	0.001447	0.010441	0.000035	0.001826	0.022329
IMI.15d.2	0.437132	0.496813	0.004273	0.003446	0.003618	0.001378	0.000724	0	0.001034	0.051583
IMI.15d.3	0.294718	0.240515	0.009028	0.036215	0.008098	0.335791	0.015472	0.002757	0.008683	0.048723
IMI.30d.1	0.025878	0.585025	0.163571	0.015024	0.006030	0.044864	0.078495	0	0.006547	0.074567
IMI.30d.2	0.574791	0.148996	0.089625	0.056201	0.038317	0.00379	0.006271	0	0.005823	0.076186
IMI.30d.3	0.577685	0.055891	0.066745	0.005582	0.024189	0.001723	0.003032	0	0.002584	0.262568

Table S5 Statistical analysis of the composition of TOP10 dominant midgut bacterial genera among different groups by one-way ANOVA (SPSS. 16.0).

**Taxonomy**	**Group**	**F**	**df**	**P**
Commensalibacter	CK.15d, T1.15d, T2.15d, T3.15d, IMI.15d	0.764	14	0.572
	CK.30d, T1.30d, T2.30d, T3.30d, IMI.30d	0.672	14	0.626
Snodgrassella	CK.15d, T1.15d, T2.15d, T3.15d, IMI.15d	0.824	14	0.539
	CK.30d, T1.30d, T2.30d, T3.30d, IMI.30d	0.488	14	0.745
Lactobacillus	CK.15d, T1.15d, T2.15d, T3.15d, IMI.15d	2.544	14	0.105
	CK.30d, T1.30d, T2.30d, T3.30d, IMI.30d	2.529	14	0.107
Gilliamella	CK.15d, T1.15d, T2.15d, T3.15d, IMI.15d	6.753	14	0.007
	CK.30d, T1.30d, T2.30d, T3.30d, IMI.30d	0.718	14	0.598
Saccharibacter	CK.15d, T1.15d, T2.15d, T3.15d, IMI.15d	1.044	14	0.432
	CK.30d, T1.30d, T2.30d, T3.30d, IMI.30d	0.398	14	0.806
Frischella	CK.15d, T1.15d, T2.15d, T3.15d, IMI.15d	0.651	14	0.639
	CK.30d, T1.30d, T2.30d, T3.30d, IMI.30d	1.029	14	0.438
Bartonella	CK.15d, T1.15d, T2.15d, T3.15d, IMI.15d	0.977	14	0.462
	CK.30d, T1.30d, T2.30d, T3.30d, IMI.30d	0.425	14	0.788
Citrobacter	CK.15d, T1.15d, T2.15d, T3.15d, IMI.15d	0.736	14	0.588
	CK.30d, T1.30d, T2.30d, T3.30d, IMI.30d	1.049	14	0.430
Bifidobacterium	CK.15d, T1.15d, T2.15d, T3.15d, IMI.15d	0.736	14	0.675
	CK.30d, T1.30d, T2.30d, T3.30d, IMI.30d	0.678	14	0.623
other	CK.15d, T1.15d, T2.15d, T3.15d, IMI.15d	1.604	14	0.248
	CK.30d, T1.30d, T2.30d, T3.30d, IMI.30d	0.887	14	0.506

Table S6 Richness estimator of 30 samples.

Sample	Observed_ species	Shannon	Simpson	Chao1	ACE	Goods_ coverage
CK.15d.1	152	2.970	0.781	209.188	233.714	0.998
CK.15d.2	214	4.395	0.884	273.368	298.542	0.998
CK.15d.3	261	4.246	0.885	299.333	300.786	0.998
CK.30d.1	174	2.916	0.725	198.391	198.810	0.999
CK.30d.2	196	3.443	0.843	281.200	288.117	0.998
CK.30d.3	258	3.632	0.862	323.833	345.445	0.997
T1.15d.1	121	2.417	0.728	168.000	177.832	0.998
T1.15d.2	162	2.310	0.590	183.969	196.860	0.999
T1.15d.3	149	2.425	0.647	169.000	166.604	0.999
T1.30d.1	192	3.477	0.850	253.034	258.909	0.998
T1.30d.2	184	1.958	0.597	226.143	241.448	0.998
T1.30d.3	196	3.397	0.764	222.037	220.522	0.999
T2.15d.1	194	3.450	0.776	263.176	253.690	0.998
T2.15d.2	268	3.477	0.791	347.286	349.924	0.997
T2.15d.3	238	2.68	0.686	307.667	322.130	0.997
T2.30d.1	154	3.386	0.812	160.955	164.106	0.999
T2.30d.2	123	1.521	0.398	195.526	196.219	0.998
T2.30d.3	160	2.363	0.663	194.182	205.265	0.998
T3.15d.1	219	2.244	0.626	269.571	269.817	0.998
T3.15d.2	316	2.792	0.518	368.528	369.121	0.998
T3.15d.3	182	3.043	0.795	312.536	323.487	0.997
T3.30d.1	197	3.360	0.799	232.15	222.158	0.999
T3.30d.2	101	0.984	0.227	121.312	126.416	0.999
T3.30d.3	199	3.445	0.833	250.207	256.061	0.998
IMI.15d1	127	1.586	0.547	237.200	221.031	0.998
IMI.15d2	166	1.753	0.577	204.607	205.897	0.998
IMI.15d3	179	2.534	0.745	240.000	250.02	0.998
IMI.30d1	188	2.628	0.634	229.577	227.464	0.998
IMI.30d2	245	2.707	0.655	378.688	365.555	0.997
IMI.30d3	248	3.309	0.666	311.103	322.849	0.998

Table S7 Statistical analysis of richness estimator among different groups by one-way ANOVA (SPSS. 16.0).

**Richness estimator**	**Group**	**F**	**df**	**P**
Obseverd_species	CK.15d, T1.15d, T2.15d, T3.15d, IMI.15d	2.76	14	0.0878
	CK.30d, T1.30d, T2.30d, T3.30d, IMI.30d	2.42	14	0.1167
Shannon	CK.15d, T1.15d, T2.15d, T3.15d, IMI.15d	6.62	14	0.0072
	CK.30d, T1.30d, T2.30d, T3.30d, IMI.30d	0.48	14	0.7530

Table S8 ANOSIM statistical analysis among different groups at 2 sampling time, respectively.

Group	R-value	P-value
IMI.15d - T3.15d	-0.0741	0.7
T2.15d - T3.15d	-0.1111	0.8
T2.15d - IMI.15d	-0.1481	0.8
Ck.15d - T3.15d	0.1481	0.4
Ck.15d - IMI.15d	0.6667	0.1
Ck.15d - T2.15d	0.1481	0.2
T1.15d - T3.15d	0.0370	0.5
T1.15d - IMI.15d	0.0370	0.5
T1.15d - T2.15d	0.1111	0.3
T1.15d - Ck.15d	0.2593	0.3
T2.30d - T1.30d	-0.1481	0.6
IMI.30d - T1.30d	0.6296	0.1
IMI.30d - T2.30d	0.1852	0.4
T3.30d - T1.30d	-0.3704	1.0
T3.30d - T2.30d	-0.3333	1.0
T3.30d - IMI.30d	0.1111	0.2
Ck.30d - T1.30d	0.1852	0.4
Ck.30d - T2.30d	-0.1481	0.9
Ck.30d - IMI.30d	0.4444	0.1
Ck.30d - T3.30d	-0.1111	0.9

Fig. S1 Melting curve, amplification curve, and standard curve of qPCR.

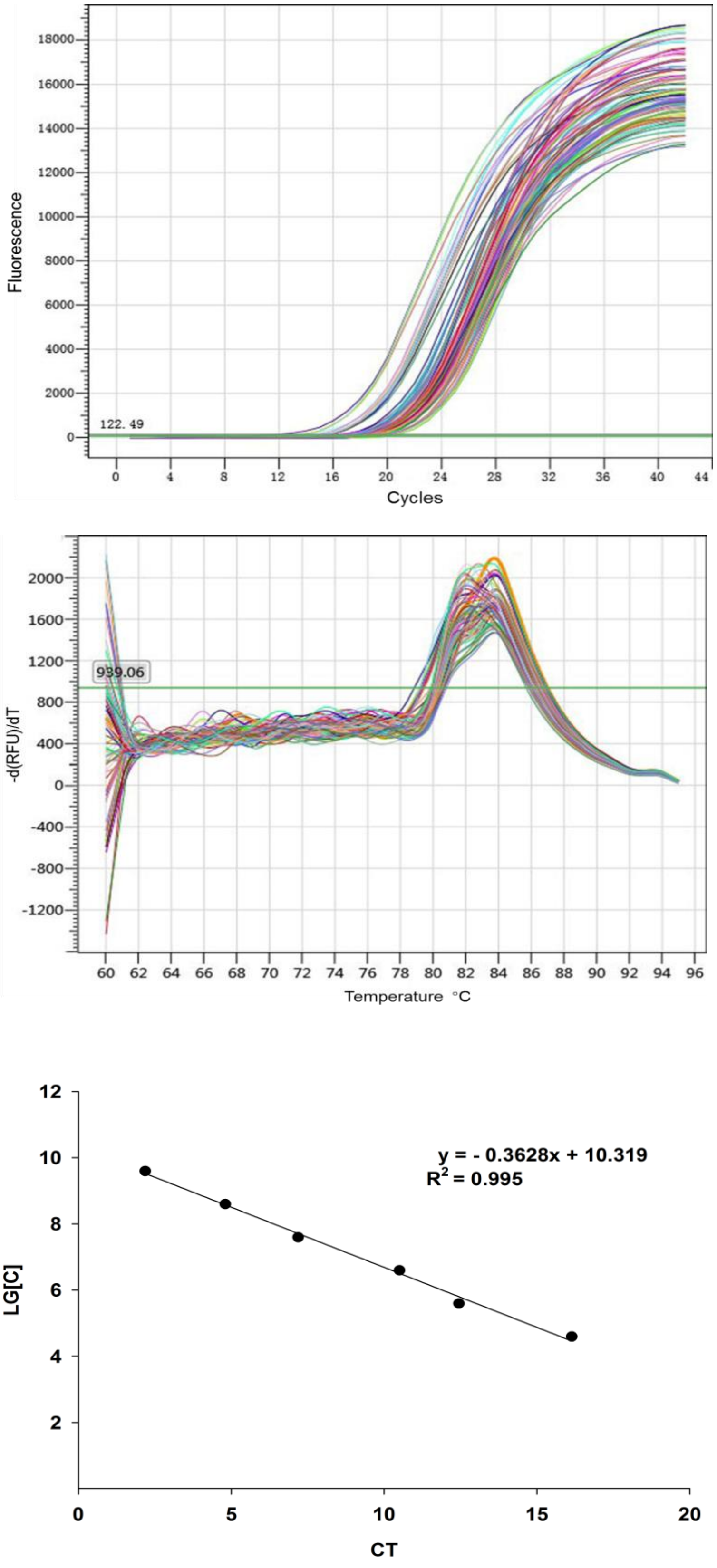

Fig. S2 Rarefaction on species-abundance data. Average value of 3 replicates were showed.

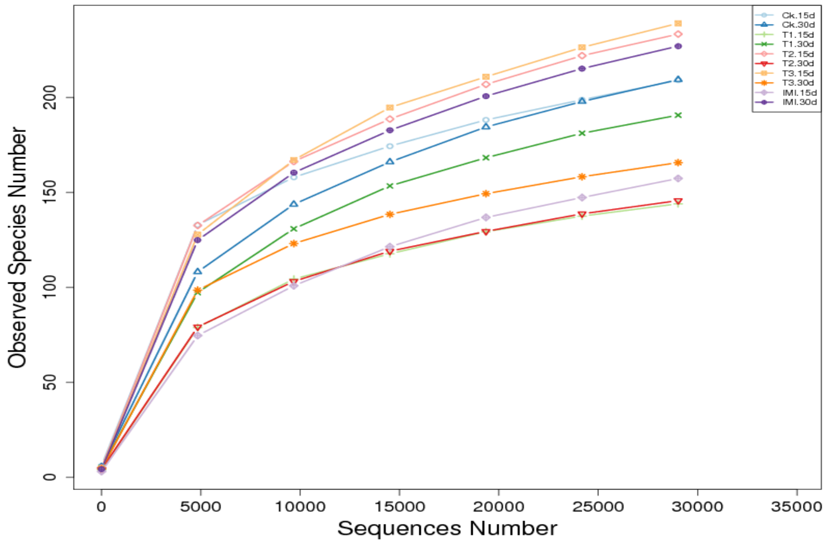

Fig. S3 Wooden cage and feeder.

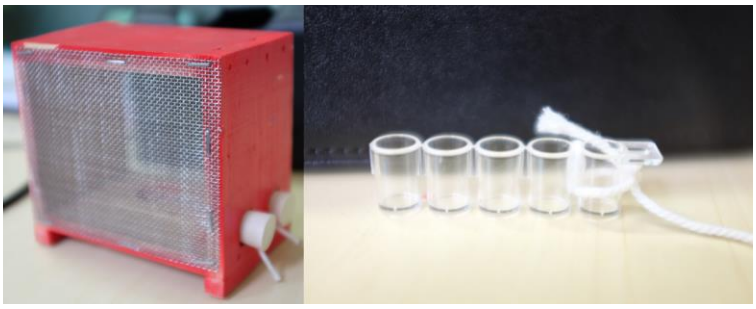

Supplement: Supplementary Information [file srep41688-s1.pdf]
